# Strict clinical and radiographic criteria for reduction of CSF shunt placement in patients with spinal myelomeningocele

**DOI:** 10.4103/1817-1745.66667

**Published:** 2010

**Authors:** Viroj Wiwanitkit

**Affiliations:** Wiwanitkit House, Bangkhae, Bangkok, Thailand - 10160

Sir,

I read the current publication of Sankhla and Khan with great interest.[1] I agree that their new criteria are successful in the reduction of cerebrospinal fluid (CSF) shunt placement. However, there are some points to be addressed. First, the clarification on the additional cost according to the protocol of new criteria should be compared to the reducing cost of reducing CSF shunt. Second, the quality of life comparing between two alternatives must be clarified. These can be useful judging the use of a new technique for reduction of CSF shunt placement
